# Radiologic-Pathologic Correlation of COVID-19-Associated Acute Disseminated Encephalomyelitis

**DOI:** 10.7759/cureus.42275

**Published:** 2023-07-21

**Authors:** Amy Bezold, Huda Hussain, Julum Nwanze, James T Roberts, Kuang-chun J Hsieh

**Affiliations:** 1 Vascular and Interventional Radiology, University of Texas Medical Branch, Galveston, USA; 2 Radiology, University of Texas Medical Branch, Galveston, USA; 3 Pathology, University of Texas Medical Branch, Galveston, USA; 4 Diagnostic Radiology, University of Texas Medical Branch, Galveston, USA; 5 Neuroradiology, Baylor College of Medicine, Houston, USA

**Keywords:** covid 19, acute disseminated encephalomyelitis (adem), mri flair sequences, atypical covid, rare cause of altered mental status

## Abstract

A 42-year-old woman presented with drooling, slurred speech, inability to walk and talk, and a recent positive COVID-19 test. She had two prior hospital admissions within the past week for similar symptoms with inconclusive evaluation. MRI of the brain demonstrated multifocal white matter hyperintense lesions on fluid-attenuated inversion recovery (FLAIR)/diffusion with variable enhancement. These imaging findings have been described in recent literature and are associated with inflammatory demyelinating disease, such as acute disseminated encephalomyelitis. The patient subsequently underwent a brain biopsy with a final diagnosis of inflammatory demyelinating lesion. To our knowledge, this is the first radiologic-pathologic correlation of COVID-19-associated acute disseminated encephalomyelitis.

## Introduction

As early as February 2020, neurological complications of SARS-CoV-2 (COVID-19) as minor as mild headache and loss of taste and smell to the more serious symptoms of acute encephalitis and seizure were documented in China [1]. A literature review a few months later suggested that central nervous system involvement of the virus stems from inflammatory complications, while peripheral nervous system complications are from immune-mediated complications [2]. Closely related to COVID-19, Middle Eastern respiratory syndrome coronavirus (MERS-CoV) and severe acute respiratory syndrome coronavirus (SARS-CoV) have been detected in brain tissue cultures, supporting the suspicion that COVID-19 has significant involvement in neuro-tissue [3-5]. For neuro-tissue, coronaviruses principally infect nuclei in the brain stem involved in cardio-respiratory balance [6]. Both SARS-CoV and MERS-CoV were additionally found to use the angiotensin-converting enzyme 2 receptors, found in most tissues, as entry into the host [7]. SARS-CoV has also been shown to infect neural tissue and, potentially through the involvement of interferons, cause inflammatory damage and neurological symptoms [4].

Mao et al. reported on the incidence of neurological symptoms of COVID-19 in early 2020. They found that 36.4% of all patients had neurological presentations. Interestingly, patients with severe infections had not only a higher incidence of impaired consciousness, at 14.8% compared to 2.4% in non-severe, but fewer of the typical symptoms such as fever and cough [6]. Reports of patients with encephalopathy were noted early, as a small minority presented with delirium, confusion, depressed consciousness, and even coma. Potential causes of the encephalopathic presentation in certain patients include spontaneous brain hemorrhages, seen in patients who are severely ill with a higher predisposition to coagulopathy, acute necrotizing encephalopathy, thought to be from a cytokine storm, and acute disseminated encephalomyelitis (ADEM), which we believe to be responsible for our patient’s presentation.

ADEM is a demyelinating disease that typically emerges in the pediatric population after a febrile, viral process or occasionally post-vaccination. Magnetic resonance imaging (MRI) on T2-weighted images and fluid-attenuated inversion recovery (FLAIR) sequences revealing diffuse or multifocal lesions in the white matter in the setting of an encephalopathic presentation supports the diagnosis of ADEM [8]. ADEM can be further complicated by necrotic findings and hemorrhagic foci that are thought to have a direct relation to COVID-19 [9]. Overall, the prognosis is poor, and early detection and initiation of treatment may improve the course of the disease. Here, we present a patient, a Texas inmate, with an encephalitic presentation who was found to have imaging findings characteristic of ADEM with confirmation upon brain biopsy in the setting of COVID-19.

## Case presentation

A non-COVID-19-vaccinated 42-year-old woman and Texas inmate was transferred to our hospital on June 23rd with a 7-day history of drooling, slurred speech, inability to walk and talk, and a subsequent positive COVID-19 test that was obtained after admission. Her neurological symptoms began as early as June 1st, starting with blurry vision and a headache that progressively worsened and was unresponsive to non-steroidal anti-inflammatory drugs. There was no history of other COVID-19 symptoms such as fevers, shortness of breath, chills, muscle pain, sore throat, new loss of taste or smell, congestion, runny nose, nausea, vomiting, or diarrhea.

Initial evaluation was performed at the outside hospital, including a magnetic resonance imaging (MRI) of the brain with limited sequences and without contrast, which showed cystic or necrotic lesions in the supratentorial compartment, primarily in the subcortical and deep white matter. After a preliminary workup, the patient was transferred to our hospital.

**Figure 1:**
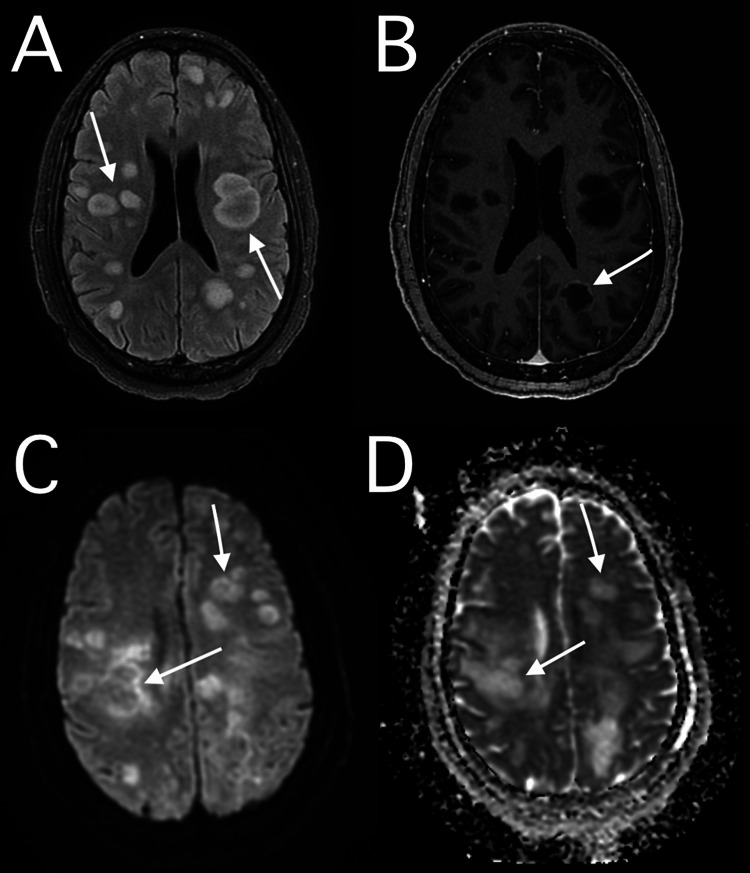
Magnetic Resonance Imaging (MRI) of the Brain MRI of the brain was performed with and without contrast which showed multifocal and confluent hyperintense lesions (arrows) in the bilateral subcortical and deep white matter on T2-weighted fluid-attenuated inversion recovery phase (A). On the T1-weighted post-contrast sequence (B), these lesions showed interrupted peripheral enhancement (arrow). Diffusion-weighted images (C) with corresponding apparent diffusion coefficient map (D) showed central elevation of diffusion coefficient with a peripheral ring of reduced diffusion coefficient (arrows).

On arrival at our hospital, she had a low-grade fever and leukocytosis with increased granulocytes. Physical examination revealed left upper extremity decorticate posturing to noxious stimuli and positive Babinski reflex. Subsequent MRI of the brain with and without contrast was performed and showed multifocal and confluent T2-weighted hyperintense lesions in the bilateral subcortical and deep white matter (Figure 1).

**Figure 2:**
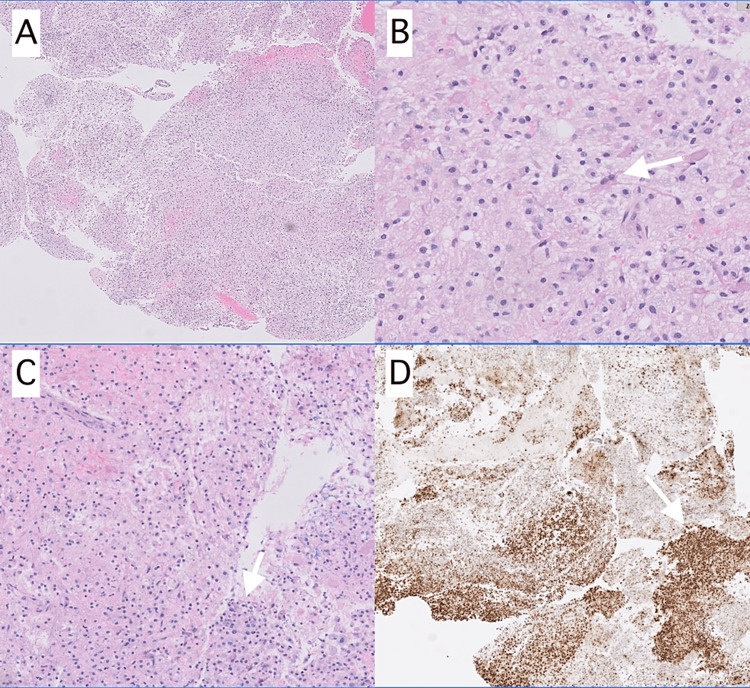
Histologic Slides of the Brain Biopsy Microscopic pathology slides were stained with hematoxylin and eosin (H&E) (A, B, and C), and CD68 (D). These slides demonstrate large numbers of discohesive clear to foamy histiocytes (arrows) and a smaller number of hypertrophic astrocytes with areas of loss of myelin, consistent with an inflammatory demyelinating lesion.

The patient was intubated on day 1 due to the development of tongue swelling and concerns for airway preservation. While many other possibilities were considered, acute disseminated encephalomyelitis or septic emboli caused by COVID-19 were strongly suspected to be responsible given the MRI findings. She started remdesivir on day 2 of admission and later, on day 6 of admission, intravenous immunoglobulin (IVIG) was initiated. Given the patient’s atypical presentation, a brain biopsy of the superficial right frontal lesion was performed on day 12 of admission with results consistent with an inflammatory demyelinating lesion (Figure 2). The pathologic findings of demyelinating lesions with perivascular infiltrates that consist mostly of foamy macrophages in combination with the previously described imaging findings favored ADEM. 

On hospital day 30, approximately 24 days after IVIG therapy, a repeat MRI of the brain with and without contrast showed similar imaging characteristics of the lesions, which were more conspicuous than the prior MRI. On day 34 she was weaned off the paralytic and began following simple commands. Further improvement in consciousness and alertness allowed cessation of steroids. On day 64 of the hospital stay, she was discharged back to her Texas Department of Criminal Justice unit.

## Discussion

The common and highly screened-for presentations of COVID-19 infection include disruptions in olfactory and gustatory sensation, fever, and the characteristic lower respiratory distress. We report on a patient who, except for a chronic headache, did not have any of the common symptoms in a COVID-19 screening. Most patients with COVID-19 who undergo neuroimaging show no specific imaging findings [10-12]. However, similar findings to our patient’s presentation, imaging, and eventual ADEM diagnosis have been reported in other hospitals, including one patient in Connecticut, who presented initially with dyspnea, fever, and vomiting, and then later in the hospital course, was found to be unresponsive with impaired oculocephalic response, flaccid muscle tone, and depressed deep tendon reflexes. This patient’s MRI revealed hyperintense lesions on FLAIR imaging, like our patient and characteristic of ADEM. She was treated with steroids and IVIG and eventually regained orientation; however, a repeat MRI found more FLAIR lesions [13]. 

The diagnosis of ADEM was supported by the results from the brain biopsy. For most cases, is not possible to distinguish between ADEM and multiple sclerosis (MS) on pathology alone, where the characteristic findings of these demyelinating diseases include demyelinating lesions with perivascular infiltrates that consist mostly of foamy macrophages [14]. However, the combination of the clinical presentation, imaging findings on MRI, and biopsy results all strongly supported the diagnosis of ADEM. While other patients have had similar presentation and imaging findings, to our knowledge, none have published corresponding pathology of these lesions. Our case is unique in this manner and helps to further support the ADEM diagnosis.

The pathophysiology of ADEM in the setting of COVID-19 infection is not fully understood, however, the mechanism of other settings of ADEM including molecular mimicry, direct central nervous system infection with an inflammatory cascade, and bystander activation could be explored [15]. High-dose steroids, intravenous immunoglobulins, and plasma exchanges have been a frequent treatment for suspected ADEM in the setting of COVID-19, as they have been shown to promote better long-term outcomes in non-COVID-19-related cases of ADEM [9,13,16]. Although the data is limited to the outcomes or treatment of ADEM in suspected COVID-19 infection, these same treatments have been the standard and were used on our patient.

## Conclusions

Neurological manifestations of COVID-19 are uncommon, and rarely lead to hospital admission. The associated neurologic imaging and pathologic findings of COVID-19 are not well documented and reported. ADEM is thought of as a disease primarily affecting the pediatric population after vaccination or viral prodrome and not in adult patients with a COVID-19 infection. ADEM should be considered in patients with rapid neurological decline with imaging findings of inflammatory demyelinating lesions in the brain and corresponding COVID-19 infection, with or without respiratory involvement.
